# Clinical Properties and Non-Clinical Testing of Mineralocorticoid Receptor Antagonists in In Vitro Cell Models

**DOI:** 10.3390/ijms25169088

**Published:** 2024-08-22

**Authors:** Luka Varda, Robert Ekart, Mitja Lainscak, Uroš Maver, Sebastjan Bevc

**Affiliations:** 1 Department of Dialysis, Clinic for Internal Medicine, University Medical Centre Maribor, Ljubljanska ulica 5, 2000 Maribor, Slovenia; luka.varda@gmail.com (L.V.); robert.ekart2@guest.arnes.si (R.E.); 2 Department of Pharmacology, Faculty of Medicine, University of Maribor, Taborska ulica 5, 2000 Maribor, Slovenia; uros.maver@um.si; 3 Division of Cardiology, Murska Sobota General Hospital, Ulica Dr. Vrbnjaka 6, 9000 Murska Sobota, Slovenia; mitja.lainscak@guest.arnes.si; 4 Faculty of Medicine, University of Ljubljana, Vrazov trg 2, 1000 Ljubljana, Slovenia; 5 Institute of Biomedical Sciences, Faculty of Medicine, University of Maribor, Taborska ulica 8, 2000 Maribor, Slovenia; 6 Department of Nephrology, Clinic for Internal Medicine, University Medical Centre Maribor, Ljubljanska ulica 5, 2000 Maribor, Slovenia

**Keywords:** mineralocorticoid receptor antagonists, spironolactone, eplerenone, finerenone, in vitro

## Abstract

Mineralocorticoid receptor antagonists (MRAs) are one of the renin–angiotensin–aldosterone system inhibitors widely used in clinical practice. While spironolactone and eplerenone have a long-standing profile in clinical medicine, finerenone is a novel agent within the MRA class. It has a higher specificity for mineralocorticoid receptors, eliciting less pronounced adverse effects. Although approved for clinical use in patients with chronic kidney disease and heart failure, intensive non-clinical research aims to further elucidate its mechanism of action, including dose-related selectivity. Within the field, animal models remain the gold standard for non-clinical testing of drug pharmacological and toxicological properties. Their role, however, has been challenged by recent advances in in vitro models, mainly through sophisticated analytical tools and developments in data analysis. Currently, in vitro models are gaining momentum as possible platforms for advanced pharmacological and pathophysiological studies. This article focuses on past, current, and possibly future in vitro cell models research with clinically relevant MRAs.

## 1. Introduction

Mineralocorticoid hormones are endogenous corticosteroids that represent an indispensable part of steroid function in the human body. The most important mineralocorticoid hormone is aldosterone, which primarily regulates sodium and potassium concentrations, with a downstream effect on water homeostasis and, finally, blood pressure [1]. It binds to the mineralocorticoid receptors in the kidney and colon epithelial cytosol, which translocates to the cell nucleus to modulate aldosterone-specific gene expression that causes sodium (re)absorption and potassium secretion. Via osmosis, water follows sodium retention and therefore regulates blood volume and, effectively, blood pressure [2]. At first, it was believed that aldosterone was produced only in the mitochondria of *zona glomerulosa* cells in the adrenal gland [3]. Only later was it suggested that the production is present in primary lymphatic organs, the intestinal tract, skin, brain, and heart [4]. The renin–angiotensin–aldosterone system (RAAS), which activates aldosterone in the end stage, is regulated by renal perfusion pressure through a baroreceptor mechanism located in the afferent arteriole of the glomerulus. Drivers for regulation are differences in chloride concentrations within the *macula densa* of the distal tubule, sympathetic nerve stimulation through β-1 receptors, and negative feedback of angiotensin II on juxtaglomerular cells [5]. A decrease in flow rate and sodium concentration is picked up by juxtaglomerular “sensory” cells, which release the enzyme renin, responsible for converting angiotensinogen into angiotensin I. With the help of another enzyme, the angiotensin-converting enzyme (ACE), angiotensin I is converted to angiotensin II. The latter, among its other functions (e.g., direct vasoconstriction via angiotensin II receptors (AT-R), stimulation of sympathetic nerve activity, vasopressin release, etc.), stimulate the production and release of aldosterone [4,6]. While the RAAS is the most important mechanism of aldosterone activation, there are alternative activation routes independent of the RAAS. Firstly, a small increase in extracellular potassium can activate aldosterone secretion [7]. Secondly, adrenocorticotropic hormone (ACTH) mostly controls glucocorticoid production that can stimulate aldosterone production via cAMP-mediated pathways and protein synthesis-independent mechanisms involving macrophage-derived factors, steroidogenesis-inducing protein and calmidazolium [8]. Thirdly, several fatty acids have the potential to stimulate aldosterone production [9]. Lastly, endothelin, vasopressin, and serotonin can also activate the aldosterone pathway, but their exact physiological role remains unclear [10].

Dysregulation and chronic activation of the RAAS can lead to chronic heart failure [11], arterial hypertension [12], endothelial dysfunction [13], prothrombotic events [14,15], and the progression of chronic kidney disease [16]. RAAS-independent mechanisms of aldosterone activation can play a pathophysiologic role in diseases such as salt-sensitive hypertension [17]. It has been shown that in salt-sensitive Dahl rats, the level of aldosterone decreased after salt loading. However, hypertension developed through renal MR activation. The proposed mechanism is the interaction between MRs and Rac1, a member of the Rho family GTPases [18].

High levels of angiotensin II and aldosterone are associated with chronic inflammation, fibrosis, and cell hypertrophy, leading to remodulation and dysfunction of cardiovascular and kidney tissue. In recent years, a link between the RAAS and bone metabolism has also been shown. RAAS components have been found in bone tissue, stimulating osteoclast formation and inhibiting osteoblasts, and therefore inducing bone loss [19].

**Figure 1:**
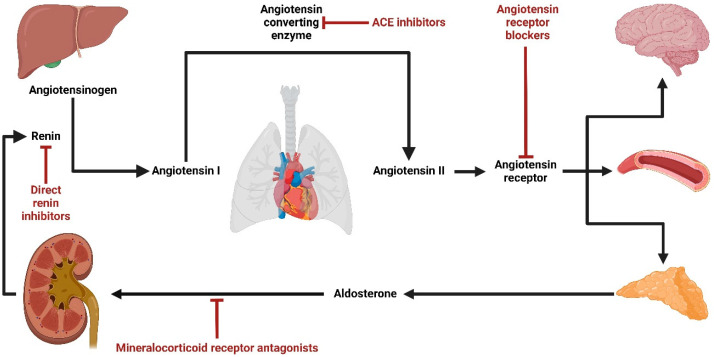
The renin–angiotensin–aldosterone system (RAAS) and effector sites of RAAS inhibitors—direct renin inhibitors, angiotensin-converting enzyme (ACE) inhibitors, angiotensin receptor blockers, and MRAs (created with BioRender.com).

Due to this, several drugs lowering the activity of the RAAS (i.e., RAAS inhibitors) were developed and have been used for many years to prevent the events mentioned above (Figure 1) [12,20].

Mineralocorticoid receptor antagonists (MRAs) inhibit the RAAS at its most distal part. Over 60 years ago, the first steroidal MRA spironolactone was introduced. Approximately 40 years later, a new class agent eplerenone proved its clinical efficacy associated with fewer adverse effects due to its higher affinity towards MRs [21]. Both drugs are used to treat chronic heart failure, resistant arterial hypertension, and hyperaldosteronism. However, their clinical use is still limited, with adverse effects like renal function worsening and hyperkalaemia, particularly when used over a longer period [22]. Finerenone is a new and emerging MRA that was introduced only recently. It presents one of the first non-steroidal MRAs and the first to demonstrate clinical efficacy with a high level of specificity [23,24]. Finerenone is currently indicated for treating diabetic kidney disease, while it is also evaluated for treating chronic heart failure [25,26]. Another non-steroidal MRA that has also reached clinical practice is esaxerenone, which was approved for the treatment of arterial hypertension in Japan and is under investigation for the treatment of diabetic kidney disease [27,28,29]. In non-clinical models, esaxerenone showed higher selectivity for MRs and was shown to inhibit MR-induced transcription more potently than spironolactone and eplerenone [30]. Due to its different molecular structure and specificity, it remains under intensive investigation. While an important part of non-clinical drug testing is still conducted in animal models [31], researchers are constantly developing novel tools to further understand drug efficiency and safety [32]. A new generation of models also includes in vitro kidney models for testing nephrotoxicity and other pharmacological effects of upcoming drugs used in treating kidney-related diseases [33]. In this mini review, the clinical properties of the general population and dialysis patients, as well as the role of in vitro cell models in the testing of MRAs will be discussed.

## 2. Methods

**Figure 2:**
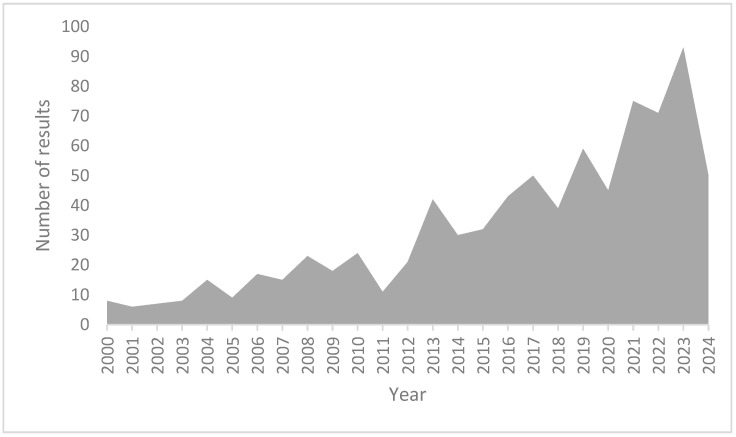
Number of results per year in ScienceDirect database search with keywords “in-vitro” and “mineralocorticoid receptor antagonist” conducted in March 2024.

Online databases PubMed, Google Scholar, and ScienceDirect were searched for relevant articles regarding the clinical properties of MRAs and in vitro cell culture models where MRAs were tested. Basic search terms included “in-vitro” and “mineralocorticoid receptor antagonist”. The search was further expanded with keywords “cell model”, “spironolactone”, “eplerenone”, and “finerenone”. This mini review therefore covers and discusses three of the most relevant MRAs and the role of in vitro models in testing these drugs on a non-clinical level. Figure 2 displays our search of the literature in the ScienceDirect database with the number of results per year.

## 3. Overview of the Current Most Commonly Used Mineralocorticoid Receptor Antagonists (MRAs)

### 3.1. Spironolactone

Spironolactone binds to cytoplasmic mineralocorticoid receptors and prevents the function of aldosterone. By its pharmacological activity, it acts as a »potassium-sparing« diuretic at the level of the distal renal tubule. Clinical indications for its use are resistant arterial hypertension, primary hyperaldosteronism, and hypervolemia due to nephrotic syndrome or liver cirrhosis [34]. It also represents one of the four pillars of the treatment of heart failure with reduced ejection fraction, alongside angiotensin receptors and neprilysin inhibitors (ARNI) or ACE inhibitors (ACEi), β receptor blockers (BB), and sodium–glucose transporter 2 (SGLT2) antagonists [35]. Contraindications are mainly acute kidney injury, hyperkalemia, anuria, and Addison’s disease. Caution is advised with the simultaneous use of potassium, including supplements and drugs that increase serum potassium levels (ACEi, renin inhibitors, angiotensin receptor blockers (ARB), non-steroidal anti-inflammatory drugs (NSAID), etc.). Therefore, in cases of hyperkalemia, spironolactone is frequently excluded from therapy. However, novel potassium binders, such as cation-exchange polymer patiromer and non-polymeric crystalline compound sodium zirconium cyclosilicate, enable its further use in cases of mild hyperkalemia [36].

Because spironolactone acts as steroidal and less-specific MRA, it also binds to androgen and, to a lesser extent, estrogen and progesterone receptors. This effect can be helpful in the treatment of acne and hirsutism [37]. On the other hand, it disrupts the menstrual cycle, and leads to gynecomastia, breast pain, impotence, and loss of libido. Other possible adverse effects include hyponatremia, nausea, vomiting, diarrhoea, headaches, and confusion. Hepatotoxic liver injury with elevated liver transaminases and alkaline phosphatase has also been described. It usually resolves with discontinuation of the drug [34,38].

End-stage renal disease (ESRD) patients are a specific subgroup of patients with chronic kidney disease (CKD). Due to complex pathophysiology and advanced kidney failure which interferes with virtually all pharmacological agents, their treatment remains a challenge. While one of the advantages of MRAs is to slow the progression of CKD to ESRD, it is also very appealing to introduce the drugs to dialysis patients, mainly due to their effects on the progression of heart failure, left ventricular (LV) hypertrophy, and hypertension. The main concerns are hyperkalemia [39] and CKD progression, yet effective therapies are in sight.

In a randomized placebo-controlled trial by Taheri et al. [40], 16 hemodialysis (HD) patients with heart failure and left ventricular ejection fraction (LVEF) < 45%, were given 25 mg of spironolactone after a regular dialysis session three times per week (*n* = 8), which significantly increased LVEF, while LV mass was significantly reduced. Both groups started with a mean serum potassium of less than 5.5 mmol/L; during follow-up, mean potassium was higher in the spironolactone group, but without a significant effect on hyperkalemia incidence [40].

Feniman-De-Stefano et al. [41] conducted a randomized, placebo-controlled trial in 17 HD patients without heart failure: spironolactone (*n* = 8) was initiated as 12.5 mg daily, which was up titrated to 25 mg daily after 1 week. LV mass was assessed using echocardiography and was significantly reduced in the spironolactone group (*n* = 8) after 6 months of treatment. Safety end-points included 24 h ambulatory blood pressure measurements and serum potassium concentrations, which were similar in both trial groups [41]. In contrast, McGill et al. [42] noted no changes in LV mass in 13 HD patients without heart failure after 9 months of spironolactone treatment at 25 mg daily [42]. Flevari et al. [43] included 14 HD patients without heart failure who received spironolactone in 25 mg doses three times per week after HD sessions. After 4 months of observation, blood pressure control, reactive hyperemia, and heart rate variability improved [43]. Vukusich et al. [44] performed a randomized, double-blind, placebo-controlled trial where the active group of non-diabetic HD patients (*n* = 33 for both groups) received spironolactone in 50 mg doses three times per week. Spironolactone significantly reduced carotid intima-media thickness after 2 years [44].

Only handful of studies reported about the spironolactone effects on survival in ESRD. Matsumoto et al. [45] conducted a trial that compared 157 HD patients treated with spironolactone at 25 mg/day and 152 control patients. The rate of cardiovascular or cerebrovascular death or hospitalization was 5.7% in the active group and 12.5% in the control group; all-cause mortality was 6.4% and 19.6%, respectively (*p* < 0.01 for all). Importantly, only three patients had to discontinue the trial due to serious hyperkalemia [45].

More than 40 years ago, Papadimitriou et al. [46] administered spironolactone at 300 mg/day to nine HD patients and recorded a significant increase in serum potassium after 3 weeks [46]. However, at low doses (<100 mg daily), many small-size studies reported spironolactone is safe in ESRD. There are currently at least two ongoing clinical trials assessing the cardiovascular outcomes of spironolactone in ESRD patients treated with HD (ALCHEMIST [NCT01848639] and ACHIEVE [NCT030203303]) [47,48].

Spironolactone has also been tested in patients on peritoneal dialysis (PD), but the data are sparse. Taheri et al. [49] conducted a randomized, placebo-controlled, double-blinded trial, and included 18 PD patients with an EF < 45%. In the spironolactone group (*n* = 9), patients received 25 mg every other day. A significant increase in EF (assessed using echocardiography) has been noted compared to the placebo group, where the EF was practically unchanged after 6 months. No significant differences were found regarding serum potassium levels between or within both groups [49]. Similarly, Ito et al. [50] included 158 PD patients, where the spironolactone group (*n* = 78) received spironolactone at 25 mg daily for 2 years. The LV mass index and LVEF improved significantly with spironolactone. Serum potassium levels were higher in the spironolactone group, but the incidence of hyperkalemia was similar in both groups [50]. Importantly, a positive effect of spironolactone on the peritoneal membrane has also been shown in PD patients [51].

### 3.2. Eplerenone

The need for a new MRA was driven by the anti-androgenic effects of spironolactone. About twenty years ago, eplerenone was produced based on its predecessor. According to its pharmacodynamic properties, it is also a competitive antagonist of aldosterone receptors. Chemically, eplerenone is characterized by the addition of a 9α,11α-epoxy bridge and the replacement of a 17α-thoacetyl group by a carbomethoxy group. Mainly, the latter resulted in a significantly lower affinity for other steroid receptors and reduced the extent of unwanted side effects [52]. Compared to spironolactone, where around 9% of patients experienced gynecomastia in the RALES trial [53], in the EMPHASIS-HF and EMPHASUS trials, the occurrence of gynecomastia was comparable between eplerenone and the placebo group [54,55]. In in vitro studies, eplerenone showed up to a 20-fold lower affinity for the aldosterone receptor than its predecessor, while in vivo studies showed that eplerenone is 50% to 75% as potent as spironolactone [56,57]. Meanwhile, the affinity of eplerenone for glucocorticoid, androgen, and progesterone receptors is 100–1000 times lower compared to spironolactone [58]. Moreover, spironolactone is metabolized into active metabolites and eplerenone into inactive ones with a shorter half-life. Both agents are included in the guidelines for their use in lowering blood pressure and treating heart failure with a reduced ejection fraction [54,55,58,59]. Eplerenone causes dose-dependent hyperkalemia, especially in patients with advanced chronic kidney disease, receiving potassium supplements and in concomitant use of »potassium-sparing« diuretics [60]. In addition, a possible increase in serum creatinine, diarrhoea, cough, fatigue, dyslipidemia, and abdominal pain have been described in the EMPHASUS trial [54]. Adverse effects of eplerenone are usually mild, with no known overdoses, except for more severe cases of hypotension and hyperkalemia [61]. The active substance cannot be dialyzed; only supportive measures are indicated [58,60].

Eplerenone was also tested in HD patients. Shavit et al. [62] included eight HD patients treated with eplerenone in 25 mg two times per day. After 4 weeks, predialysis systolic blood pressure was significantly reduced. Furthermore, changes in serum potassium were insignificant. However, eplerenone had no effect on diastolic blood pressure, predialysis weight, intradialytic weight gain, or plasma aldosterone [62]. Walsh et al. [63] tested the safety of eplerenone in HD patients. A total of 77 patients received eplerenone in 50 mg daily and were compared to 77 patients receiving a placebo and followed for 13 weeks. No significant dropout rate due to hyperkalemia or hypotension was recorded throughout the trial. However, nine patients developed hyperkalemia (>6.5 mEq/L) in the eplerenone group, compared to two patients in the placebo group [63]. A case report of eplerenone used in resistant hypertension in PD patients reported a reduction in mean ambulatory blood pressure and serum potassium within normal ranges [64].

### 3.3. Finerenone

**Figure 3:**
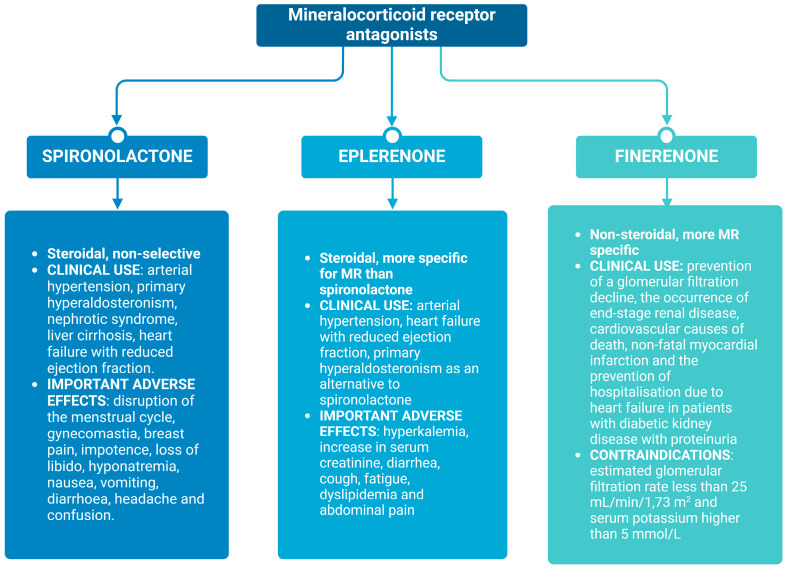
Clinical properties of most widely used mineralocorticoid receptor antagonists (created with BioRender.com).

In numerous clinical studies, spironolactone and eplerenone have shown favourable antiproteinuric effects in addition to ACEi or ARBs, especially in patients with diabetic kidney disease [65,66,67]. The goal was to produce a potent MRA, which will summarise all the favourable cardiovascular and renal effects and, simultaneously, have as few unwanted effects as possible, especially anti-androgen activity and hyperkalemia. As such, the specific and non-steroidal MRA finerenone has been an emerging example. General upsides of non-steroidal MRAs compared to steroidal MRAs are their high affinity, improved MR specificity, and a significantly better therapeutic index, which is defined as a ratio of the drug dose that causes a significant increase in serum potassium to the dose that is needed to improve clinical parameters and outcomes, for example, albuminuria [68,69,70]. Compared to steroidal MRAs, they are less lipophilic and more polar, and therefore don’t cross the blood–brain barrier. With this trait, they also have a different distribution and tissue penetration [71,72]. By binding to the MR, finerenone inhibits the recruitment of transcriptional co-factors involved in expressing hypertrophic, inflammatory, and fibrotic genes. It is at least as potent as spironolactone and more specific for MRs than both predecessors [73]. According to existing research, the new MRA is comparable to others in terms of its positive effects on the occurrence and progression of heart failure and its beneficial effect on proteinuria, as well as the progression of chronic kidney disease, especially of a diabetic aetiology. At the same time, it was shown to lead to much less unwanted side effects [74,75]. In clinical practice, it is indicated for the prevention of a glomerular filtration decline, the occurrence of end-stage renal failure, cardiovascular causes of death, non-fatal myocardial infarctions and the prevention of hospitalization due to heart failure in patients with chronic kidney disease associated with type 2 diabetes. Prescription is contraindicated in patients with a glomerular filtration rate below 25 mL/min/1.73 m^2^ and with serum potassium values above 5 mmol/L [25]. In phase III clinical trials, there was still a significantly higher incidence of hyperkalemia compared to the placebo, but discontinuation due to an adverse effect was significantly lower [76,77,78]. It has been shown that finerenone has no active metabolites and a shorter half-life than steroidal MRAs, which could, alongside more balanced tissue distribution, contribute to a lower risk of hyperkalemia [72,79]. Despite all the advantages of finerenone, the original MRAs are still more effective in lowering blood pressure and for use in resistant arterial hypertension [78,80]. To the best of our knowledge, finerenone has not yet been tested in PD or HD patients in a clinical trial. However, studies are ongoing—patients included will be on HD for at least 3 months, on therapy with ACEi or ARB at maximum tolerable doses, with serum potassium ≤ 5.7 mmol/L, LVEF ≤ 50%, and a pulse wave velocity higher than age-estimated values [81]. Important clinical properties of these three MRAs are presented in Figure 3.

## 4. MRA Testing on In Vitro Cell Models

**Figure 4:**
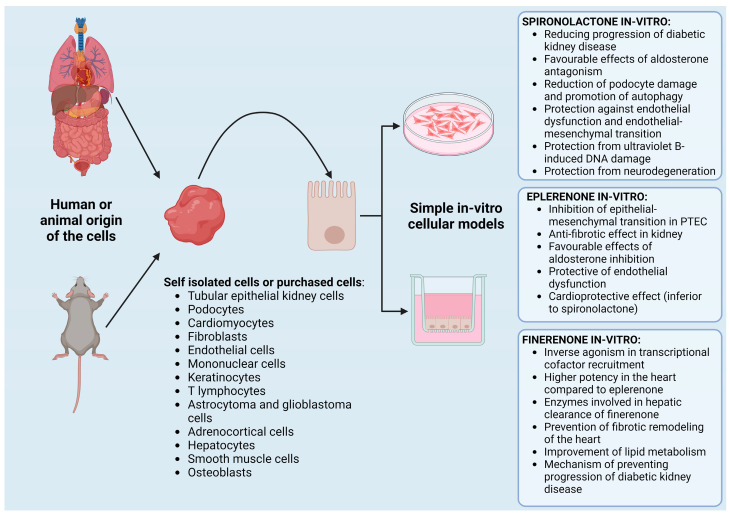
Summary of in vitro cellular models used for the testing of spironolactone, eplerenone, and finerenone (created with BioRender.com). PTEC—proximal tubular epithelial cell.

The idea of non-clinical drug testing on in vitro cellular models originates from animal studies failing to predict human responses to drugs precisely enough [31]. Regarding toxicological studies, single-cell-type models have already shown many promising results [82]. However, since they include only one cell type and lack the in vivo tissue complexity (e.g., 3D structure, extracellular matrix components, less relevant physiological communication), they often fail to mimic the in vivo tissue closely enough and tend to cause the cells to lose their shape and phenotype [83]. Therefore, single- and multi-layer co-cultures of different cell types, organoids and »organs-on-a-chip« have been created to upgrade the original model’s complexity. The latter represents the most complex cellular model created to this day, enabling the creation of a multicellular machine with autocrine and paracrine cell-to-cell communication, perfusion, and mechanical stress imitation, which provides more prolonged cell survival and, therefore, more extended experiments [33,84]. The cells used in the models can be obtained from animals or humans. Such models commonly use immortalized cell lines with their advantages and drawbacks [33,85,86]. Non-clinical tests for MRAs on these models are discussed below (Figure 4).

### 4.1. In Vitro Testing of Spironolactone

Rat renal tubular epithelial cells (NRK-52E) were cultured in in vitro conditions and exposed to different glucose concentrations. High glucose increased the expression of Toll-like receptor 4 (TLR4) and inflammatory parameters in cells, suggesting a potential role of TLR4 in the progression of diabetic kidney disease [87]. Spironolactone reduced the expression of TLR4 and inflammatory mediators in vitro. With a simple cell culture, one of the protective mechanisms of spironolactone against the progression of diabetic kidney disease was thus presented [87]. Furthermore, the cultivation of immortalized human proximal tubular epithelial kidney cells (PTEC) and rat fibroblasts in monoculture showed that the RAAS inhibitors (including MRAs) directly reduce renal fibrosis caused by elevated blood sugar levels. They did this by inhibiting the expression of platelet-derived growth factor (PDGF) and connective tissue growth factor (CTGF/CCN2) in tubular cells, which was increased in hyperglycaemic conditions. However, the complexity of the model was not very high, and the co-culture of both cell types was not created, which could improve the in vitro model and possibly better simulate in vivo processes [88].

Aldosterone caused cell damage in HK-2 proximal tubule cells by activating the mitochondrial pathway and producing reactive oxygen species. The effects created were via MR agonism, thus suggesting a potential role of MRAs in their inhibition [89]. A few more monoculture models of PTECs were developed to study the effects of aldosterone, with spironolactone acting as an antagonist and showing prevention of some of the effects of aldosterone. Still, the primary purpose of the research was not to test the effects of spironolactone [90,91,92,93,94,95].

**Figure 5:**
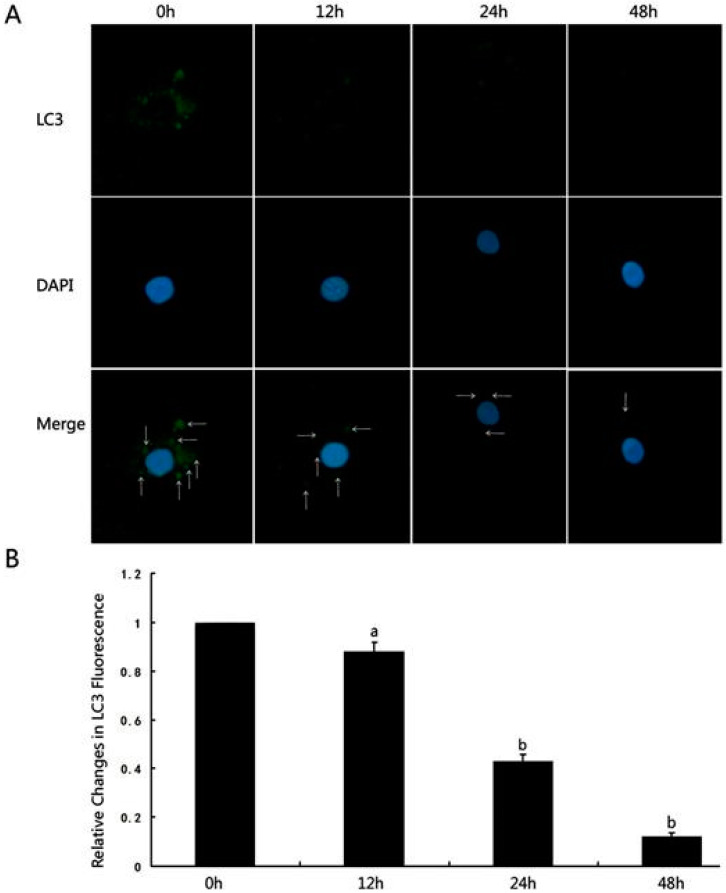
Human conditionally immortalized podocytes were put under mechanical stress. (**A**) Immunostaining for LC3 puncta and 4′,6-diamidino-2-phenylindole (DAPI)—binding to DNA and presenting nucleus was performed at 0 h, 12 h, 24 h, and 48 h and visualised under confocal microscope (magnification ×1200). LC3 dots are most prominent in the perinuclear and cytoplasmic regions (white arrows in merged images). LC3 puncta staining is lower at 12 h, 24 h, and 48 h, suggesting lower autophagy under mechanical stress. (**B**) Quantification of LC3 puncta staining showing statistically significant reduction at 24 h and 48 h (b) and statistically non-significant reduction at 12 h (a) [96]. »Reproduced with permission from Li et al., *Spironolactone promotes autophagy* via *inhibiting PI3K/AKT/mTOR signalling pathway and reduce adhesive capacity damage in podocytes under mechanical stress.* Published by Bioscience Reports—Portland Press, 2016«. The article is licensed under an open access Creative Commons CC BY 4.0 license [96].

The basic mechanisms by which spironolactone prevented the reduction in the adhesive abilities of podocytes under mechanical stress were also demonstrated in cell cultures, emphasizing autophagy [96]. Human conditionally immortalized podocytes subjected to mechanical stress were treated with spironolactone for 48 h. Immunofluorescence staining for LC3 puncta, which is a commonly used marker of autophagosomes [97], showed that the level of autophagy was reduced under mechanical stress (Figure 5). Abnormal PI3K/Akt/mTOR pathway activation was demonstrated, and integrin β1 and atg5 expression was lower. In this model, spironolactone increased the expression of integrins and decreased the expression of p85-PI3K, p-Akt, and p-mTOR, promoting autophagy and reducing podocyte damage [96].

In the culture of mononuclear cells, lipopolysaccharide (LPS) was used to stimulate the cells to produce pro-inflammatory cytokines [98]. Spironolactone lowered the accumulation of tumour necrosis factor (TNF)-α in this setting and also induced apoptosis, which was evaluated with phosphatidylserine externalisation and DNA fragmentation using annexin V/PI or terminal deoxynucleotidyl transferase dUTP nick end labelling (TUNEL) flow cytometry kits. Timewise, TNF- α suppression occurred first and apoptosis later. In contrast, the concentration of interleukin (IL)-1β increased with the addition of the drug, but only when spironolactone was added to the culture after LPS. Thus, the IL-1β may have been elevated due to previous damage to the cells caused by LPS-induced inflammation even before the addition of spironolactone. The authors failed to answer the question of how the two phenomena (inhibition of inflammatory mediators and apoptosis) are interconnected. The mechanism by which spironolactone causes apoptosis was also not elucidated [98].

To clarify the mechanism of stiff endothelial cell syndrome, cultures of human umbilical vein endothelial cells (HUVEC), bovine aorta, and human artery explants were created in an aldosterone-enriched medium with and without the addition of spironolactone [99]. MR expression, epithelial sodium channel expression, surface area, and model stiffness increased over time. Spironolactone effectively prevented the mentioned processes and increased nitric oxide (NO) release by 50% after three weeks of treatment [99]. In another study, endothelial cells obtained from the human umbilical artery were also cultured with aldosterone, with and without the addition of spironolactone [100]. The results indicated that aldosterone plays a crucial role in nicotinamide adenine dinucleotide phosphate (NADPH) oxidase-mediated oxidative stress, resulting in decreased endothelial NO synthase (eNOS) expression in human endothelial cells. Spironolactone effectively reversed these consequences, suggesting its potential protective effect in endothelial dysfunction [100]. Spironolactone was further shown to inhibit fibrosis and the process of endothelial–mesenchymal transition, demonstrated in a HUVEC culture by decreased expression of vimentin and increased expression of CD31, as well as by preventing cell migration [101].

Using human keratinocytes in vitro and skin explants ex vivo, spironolactone was found to rapidly deplete xeroderma pigmentosum type B (XPB) protein in both systems and abrogate two major responses to ultraviolet B (UVB)-induced DNA damage, including the removal of UV photo products from genomic DNA and the activation of ataxia-teleangiectasia mutated (ATM)/ATM- and Rad3-related (ATR) kinases [102]. These effects have been associated with mutagenesis and susceptibility to UVB-induced cell death and were observed only in the case of spironolactone. On the contrary, neither its metabolites, canrenone and 7α-thiomethylspironolactone, nor the more specific MRA eplerenone affected the XPB protein levels or the response to UVB [102].

Interestingly, spironolactone also inhibited human immunodeficiency virus (HIV)-1 and HIV-2 infection of T cells in vitro via antagonistic effects on Tat-dependent transcription [103]. Since Tat protein enhances viral transcription, drugs that target Tat or the cellular co-factor nucleus factor (NF)-κB seem a sensible choice in HIV infection prevention. The treatment of Jurkat and primary CD4^+^ T cells with spironolactone induced degradation of the cellular xeroderma pigmentosum B (XPB) helicase without affecting cellular mRNA levels, T cell viability, or T cell proliferation. This effect was independent of spironolactone’s aldosterone-blocking function because eplerenone did not induce XPB degradation or inhibit HIV infection [103].

Among other things, spironolactone has also shown beneficial effects in the case of neurodegenerative diseases in animal models. Cell cultures of neuroblastoma and astrocytoma cells were made to study these effects in vitro. Both were exposed to N-methyl-D-aspartate (NMDA) or chloroquine, which caused toxic damage. With the addition of spironolactone, researchers recorded a lower percentage of dead cells and increased cell viability in the case of toxic damage with NMDA and chloroquine [104].

Methylglyoxal (MG) is a reactive dicarbonyl metabolite of the glucose degradation pathway that can accumulate in patients with diabetes [105]. Park et al. [106] treated osteoblastic MC3T3-E1 cells with spironolactone in the presence of MG. It reduced endoplasmic reticulum stress, the production of intracellular reactive oxygen species, mitochondrial superoxides, cardiolipin peroxidation, and inflammatory cytokines. MG induced mitochondrial dysfunction, but markers of mitochondrial biogenesis such as mitochondrial membrane potential, adenosine triphosphate, proliferator-activated receptor gamma coactivator 1α, and nitric oxide were significantly improved by spironolactone. This in vitro study shows the potential of spironolactone and its favorable effects on bone metabolism [106].

### 4.2. In Vitro Testing of Eplerenone

**Figure 6:**
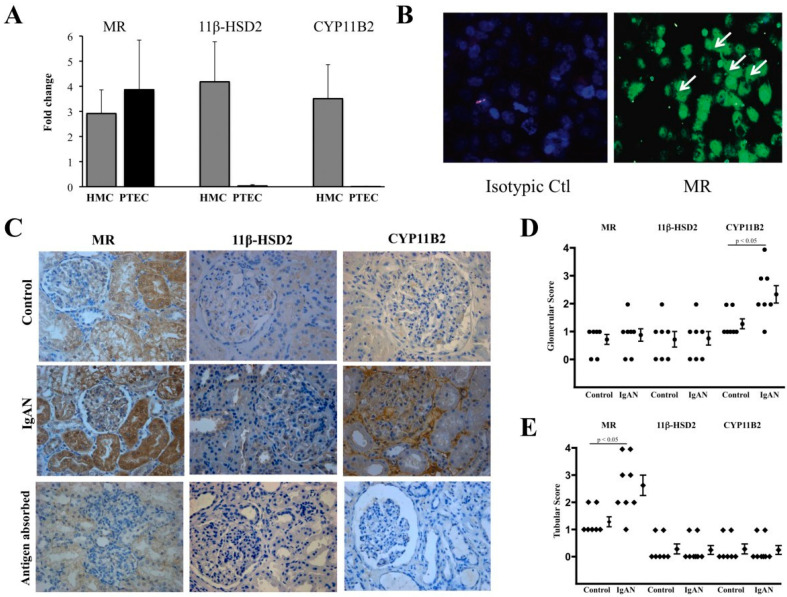
Kidney biopsies of IgA nephropathy patients were obtained, and human mesangial cells (HMC) and human PTEC of IgA nephropathy patients were cultured, and the expression of MRs, 11β hydroxysteroid dehydrogenase (11β-HSD2), and CYP11B2 were determined using qPCR and immunofluorescence. (**A**) Both cell types expressed MR mRNA, whereas HMC also expressed 11β-HSD2 and CYP11B2. (**B**) Immunofluorescence of MR expression on PTEC (arrows) under magnification ×200. (**C**) Detection of all three markers in kidney biopsies of IgA nephropathy patients and controls under magnification ×200—MRs was expressed in glomeruli and tubules, whereas the other two markers were only in glomeruli. (**D**) A five-point scale was used to show increased glomerular CYP11B2 staining in IgA nephropathy patients compared to controls. (**E**) A five-point scale was used to show increased tubular MR staining in IgA nephropathy patients compared to controls. [110]. »Reproduced with permission from Leung et al., *Oxidative damages in tubular epithelial cells in IgA nephropathy: role of crosstalk between angiotensin II and aldosterone*; published by Journal of Translational Medicine—Springer Link, 2011«. The article is licensed under an open access Creative Commons CC BY 4.0 license [110].

An inhibitory effect of eplerenone on the epithelial–mesenchymal transition was demonstrated in the immortalized human PTEC cell line in monoculture through the decreased expression of α-smooth muscle actin (α-SMA), which proves the transition of cells to myofibroblasts, and the increased expression of E-cadherin [107]. The same cell line was also used to demonstrate aldosterone activation of the Rho–kinase pathway. The latter induces the secretion of extracellular matrix components in PTEC. Eplerenone almost completely inhibited the process, demonstrating its potential anti-fibrotic property [108]. Similar to spironolactone, eplerenone was also included in studies where the primary purpose was to investigate the effects of aldosterone. At the same time, MRAs served only as a receptor blocker in part of the study [109,110,111]. In monoculture, aldosterone was shown to increase cell swelling, cell surface area, and stiffness of endothelial cells of venous origin (EAHy 926), as well as human coronary artery endothelial cells. Eplerenone reliably reduced these adverse effects and demonstrated an in vitro ability to prevent endothelial dysfunction (Figure 6) [112].

Spironolactone and eplerenone were tested in human adrenocortical H295R cell cultures expressing MRs [113]. Spironolactone inhibited the basal production of cortisol and aldosterone. After treating H295R cells with angiotensin II, which increased aldosterone production, spironolactone inhibited angiotensin II-dependent aldosterone and cortisol production. In contrast, eplerenone did not inhibit basal angiotensin II-stimulated, forskolin-stimulated or pregnenolone-stimulated cortisol production or aldosterone production. Eplerenone did lower aldosterone-stimulated MR activity, but spironolactone was more potent in this action. These data suggest that, in contrast to spironolactone, the same concentrations of eplerenone (the maximum being 30 µM) do not inhibit aldosterone or cortisol production by adrenal cells in vitro. The study fails to explain the mechanism by which the process is executed but suggests that mechanisms other than MR inhibition are involved, as concentrations needed to inhibit steroid production were higher than concentrations needed to suppress MR activity [113]. To study the cardioprotective mechanisms of MRAs, a culture of neonatal Wistar rat cardiomyocytes and fibroblasts was prepared with and without aldosterone [114]. Spironolactone and eplerenone both stimulated cardiomyocyte proliferation, even in the absence of aldosterone. Therefore, it is believed that these effects are independent of aldosterone antagonism. Spironolactone was able to reduce fibroblast proliferation and virtually abolish aldosterone-stimulated proliferation. The same, but to a lesser degree, was found for eplerenone. Overall, the study showed a slightly better in vitro cardioprotective effect of spironolactone compared to eplerenone, as well as aldosterone-independent pharmacodynamic effects of both drugs [114].

Human monocyte-derived macrophages were incubated in monoculture with aldosterone and eplerenone alone and combined with both substances [115]. A pro-inflammatory response was detected upon adding aldosterone as macrophages released molecules such as NO and oxygen reactive species. Both inducible NO synthase (iNOS) mRNA and protein changes were proportional, suggesting the action of aldosterone happened at the level of gene expression. However, the authors did not use any gene transcription or translation inhibitors to test the non-genomic effect of aldosterone. Meanwhile, eplerenone did not attenuate the response of macrophages to aldosterone and reduce the release of the above-mentioned inflammatory molecules. However, eplerenone did affect the expression of arginase I and mannose receptors at the gene expression and protein level. The latter has been shown to have a role in alternative macrophage activation, which opposes inflammation [116]. Therefore, the effect of eplerenone on macrophages could be through their alternative activation, which could prevent the release of pro-inflammatory molecules. In this study, macrophages of healthy young adults were obtained, and the treatment time with aldosterone was short [115]. A primary culture of human macrophages obtained from heart failure patients was created to study their response to aldosterone and eplerenone [117]. Compared to monocytes of healthy individuals, they had higher mineralocorticoid activity, NO and reactive oxygen species production, as well as a higher expression of iNOS. Aldosterone further increased the mentioned parameters, while eplerenone had the opposite effect. Interestingly, eplerenone attenuated mineralocorticoid activity only in macrophages obtained from heart failure patients. The combined treatment of aldosterone and eplerenone presented inverse agonism in healthy macrophages and competitive antagonism in heart failure macrophages, which could suggest that the state of heart failure changes the function of MRs or suppresses the expression of membrane receptors for aldosterone. Alternative macrophage activation performed by IL-4 was quantitatively higher in healthy macrophages, while eplerenone showed mixed activation of classical and alternative macrophage activation [117].

### 4.3. In Vitro Testing of Finerenone

**Figure 7:**
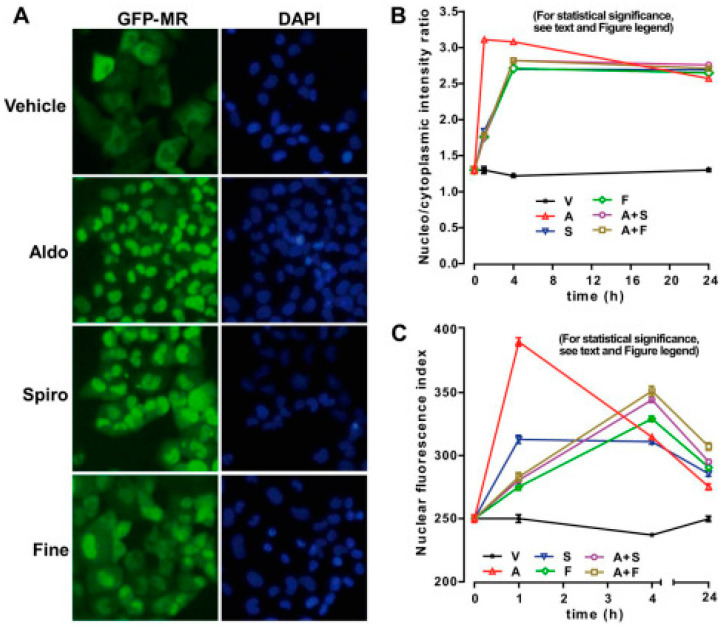
Both tested MRAs can delay MR nuclear translocation. (**A**) After culturing human kidney GFP-MR cells for 48 h, incubation with aldosterone, spironolactone, and finerenone followed. Anti-GFP antibodies were used for immunocytochemistry with automated ArrayScan VTI fluorescent microscope (left side). DAPI was used for marking the nucleus (right side)—magnification ×20. With aldosterone treatment, complete accumulation of GFP-hMR in the nucleus was seen, but not with finerenone and spironolacotne, where it was partially located in the nucleus (**B**) Translocation index (average nuclear intensity/average cytoplasmic intensity) was calculated. (**C**) Average nuclear fluorescence intensity values calculated. V—vehicle; A—aldosterone; S—spironolactone; F—finerenone; Aldo—aldosterone; Spiro—spironolactone; Fine—finerenone [124]. »Reproduced with permission from Amazit et al., *Finerenone impedes aldosterone-dependent nuclear import of the mineralocorticoid receptor and prevents genomic recruitment of steroid receptor coactivator-1*; published by Journal of Biological Chemistry—currently published by Elsevier; originally published by American Society for Biochemistry and Molecular Biology., 2015«. The article is licensed under an open access Creative Commons CC BY 4.0 license [124].

Most non-clinical studies of finerenone thus far have been conducted in animals [118,119,120,121,122]. However, additional in vitro experiments played an important role in many studies. On a rat cardiomyocyte cell line H9C2/MR^+^, expressing MR and HEK 293T kidney cells, the difference between steroidal and non-steroidal transcriptional co-factor recruitment was shown [123]. Whereas eplerenone showed partial agonistic recruitment, finerenone was an inverse agonist in vitro in the absence of aldosterone. Furthermore, in the presence of aldosterone, finerenone showed more potency and efficacy than eplerenone in inhibiting MR co-factor binding. Moreover, in the presence of a S810L mutation of MRs, which is a cause of a severe form of familial hypertension, finerenone acted as an antagonist compared to spironolactone, which was an agonist (Figure 7) [123,124]. Cardiomyocyte cell line H9c2 and neonatal rat ventricular myocytes were used to prove that finerenone can, in contrast with eplerenone, induce G protein-coupled receptor-kinase (GRK)-5-dependent cardiac MR inhibition, suggesting its higher potency in the heart [125].

In vivo and in vitro experiments were designed to determine the pharmacokinetic properties of finerenone [126]. The latter included incubation of the drug with cryopreserved human hepatocytes, showing that CYP3A4 inhibitors (erythromycin and verapamil) reduced its clearance by 77% and 87%, respectively. After adding a CYP2C8 inhibitor, the clearance of finerenone was inhibited completely. The study suggested these were the only enzymes involved in the hepatic clearance of the drug [127,128]. Furthermore, finerenone was shown to be an inhibitor of CYP1A1, CYP2C8, CYP2C9, CYP2C19, and CYP3A4 in vitro [126].

Using human coronary artery smooth muscle cells and human umbilical vein endothelial cells in vitro, it was shown that finerenone can prevent endothelial cell apoptosis and smooth muscle cell proliferation induced by aldosterone [129]. Furthermore, it reduced the inflammatory response, attenuated smooth muscle cell proliferation and neointimal lesion formation, and induced re-endothelization after vascular injury. Altogether, it shows the potential for favourable vascular outcomes and its potential use in patients with ischemic cardiomyopathy [129]. Using neonatal rat cardiac fibroblasts, finerenone has shown its ability to prevent nuclear MR translocation induced by aldosterone and decrease the expression of connective tissue growth factor, indicating fibrotic remodelling prevention [130].

A preadipocyte cell line (T37i) was differentiated into adipocytes and incubated with finerenone, showing that a MR blockade promotes adenosine monophosphate-activated protein kinase (AMPK) activation and regulates lipid metabolism [131]. In neonatal rat cardiomyocytes, finerenone also improved lipid metabolism and decreased inflammation and apoptosis after incubation in a high-glucose and fatty acid medium [132].

A human renal cortical cell line stably expressing MRs was exposed to aldosterone [133]. Analysis of the effects of spironolactone and finerenone on the transcriptome of this cell line was performed using RNA sequencing. Several genes have been identified that are either induced or repressed by aldosterone. Both MRAs were shown to inhibit aldosterone-induced and aldosterone-repressed genes, but finerenone had quantitatively more effective antagonism on some of them. It induced better inhibition of the dual specificity protein phosphatase 1 (DSUP1), hedgehog-interacting protein (HHIP), inositol hexakisphosphate kinase 3 (IP6K3), ubiquitin specific peptidase 2 (USP2), TSC domain family protein 2 (TSC22D2), period circadian protein homolog 1 (PER1), and sodium channel epithelial 1 subunit alpha (SCNN1A) genes, which were induced by aldosterone [133]. HHIP has previously been expressed in diabetic kidney disease, where it was involved in the fibrosis and apoptosis of glomerular endothelial cells [134]. Furthermore, IP6K3 has been shown to be beneficial in reducing weight gain and insulin resistance in mice [135]. On the other hand, lower DUSP1 expression was associated with glucose metabolism disorder, renal dysfunction, and fibrosis [136]. These could suggest finerenone’s involvement in the treatment and prevention of diabetic kidney disease; however, further investigation of the genes is required.

In the culture of human renal glomerular endothelial cells, it was demonstrated that at elevated glucose levels, metastasis-associated lung adenocarcinoma transcript 1 (MALAT1) activates methyltransferase G9a, raises H3K9me1, and thus epigenetically inhibits the expression of the Klotho gene and causes damage to glomerular endothelial cells. In this study, finerenone significantly inhibited the expression of MALAT1 when the cell culture was exposed to high glucose concentrations, suggesting its important role in treating diabetic kidney disease [137].

## 5. Conclusions

Spironolactone and eplerenone are already widely used MRAs, with many known clinical and side effects. Clinical testing has also been performed on dialysis patients, where the progression of heart failure and treatment of hypertension has been the main objective. The use of these drugs has been proven to be safe in low doses in this subpopulation of patients. Finerenone is joining spironolactone and eplerenone in clinical practice and is currently intended for non-dialysis patients with chronic kidney disease of diabetic aetiology. While many clinical studies are ongoing, non-clinical testing is also resuming, trying to further explain the mechanism of action and side effects of the drugs. Most non-clinical research on MRAs is still performed on animal models. However, some cellular signalling pathways, the aldosterone-inhibiting effect, and the cardio- and nephroprotective effects have also been demonstrated in in vitro cell models, despite using rather simple models, mostly in the form of monocultures of animal origin, which do not bypass the problem of cross-species differences. Nonetheless, in vitro models present a possible platform to test old and upcoming pharmacological agents. Advancements in biomedical technology enable the formation of in vitro models of different complexities, from simple single-layer monocultures to complex co-culture »organ-on-a-chip« technologies and multicellular organoids. In the future, it would be necessary to prepare more complex in vitro cell models with several types of cells in the form of co-cultures, preferably of human origin, eliminating the differences between human and animal responses to medical agents. Further molecular mechanisms of older and upcoming MRAs could be enlightened in more complex in vitro models, where in vivo tissue would be mimicked more precisely and the response to drugs could be better elucidated. Whether in vitro cell models are superior or inferior to animal studies remains the subject of further experiments.

## Data Availability

The data will be provided upon request.
